# Reconstruction of noisy images via stochastic resonance in nematic liquid crystals

**DOI:** 10.1038/s41598-019-40676-6

**Published:** 2019-03-08

**Authors:** Xingpan Feng, Hongjun Liu, Nan Huang, Zhaolu Wang, Yongbin Zhang

**Affiliations:** 1 State Key Laboratory of Transient Optics and Photonics, Xi’an Institute of Optics and Precision Mechanics, Chinese Academy of Sciences, Xi’an, 710119 China; 2 Collaborative Innovation Center of Extreme Optics, Shanxi University, Taiyuan, 030006 China; 3 University of Chinese Academy of Sciences, Beijing, 100084 China

## Abstract

We employ nematic liquid crystals as the nonlinear medium to recover noisy images via stochastic resonance, in which nonlinear coupling allows signals to grow at the expense of noise. The process is theoretically analyzed and the cross-correlation is numerically calculated. It is found that the quality of output images is affected by the input noise intensity, the applied voltage and the correlation length of noise light. Noise-hidden images can be effectively recovered by optimizing these parameters. The results suggest that nematic liquid crystals can be used for reconstruction of noisy images via stochastic resonance based on modulation instability with molecule reorientation nonlinearity.

## Introduction

For optical imaging, strong noise often submerges a low-level signal, which makes it difficult to be distinguished. In linear systems, noise is considered harmful to signal in general. In nonlinear systems, the role of noise is complicated. A particular level of noise can make signal enhanced, which is named as stochastic resonance (SR)^1^. Many researches of SR have been made from climatic change to electrical systems and biology^2–4^. In Recent years, this effect attracts considerable attention in optical fields, but most works are made by exploiting the bistability of a medium or system^5,6^. Particularly, Sharpe *et al*. observed SR based on the bistability of aligned ferroelectric liquid crystal and investigated SR in a two-dimensional array based on the bistability created by a liquid crystal light valve in an optical feedback loop^7,8^. For extracting two-dimensional images, Dylov *et al*. have demonstrated a new type of SR with the nonlinear coupling between coherent signal and incoherent noise based on modulation instability in the photorefractive crystal. This effect can be used to reconstruct images without detector threshold or feedback^9,10^. Han *et al*. developed and proved the model for reconstruction of nanosecond pulse images theoretically and experimentally^11,12^. Zhang *et al*. established a simple and intuitional particle model to simulate nonlinear coupling in a photorefractive crystal by treating light as moving particles^13^.

In this paper, we apply nematic liquid crystals (NLCs) as the nonlinear medium in a nonlinear system similar to the one in reference^9^ and study reconstruction of noisy images via SR. Unlike researches in reference^7,8^ where SR is based on the bistability to process signal in time domain, SR in our study is based on modulation instability with molecule reorientation nonlinearity in NLC to recover noisy images and our system does not need feedback or to apply noise in the form of voltage. NLC has the mature processing technology and is convenient to adjust with electricity or magnetism. It will be a better medium for the large-scale practical application of noisy images reconstruction via SR because of the above advantages. At the same time, the results can give an insight into the mechanism of noisy images recovery via SR in a high-nonlocal nonlinear medium like NLC.

## Results and Discussion

### Schematic diagram and design

**Figure 1:**
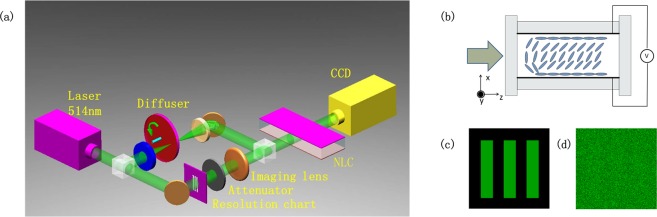
(**a**) Schematic diagram to reconstruct noisy image via stochastic resonance using nematic liquid crystals. NLC: nematic liquid crystal. (**b**) Sketch of NLC cell. Cell thickness across *x* axis is 330 μm. Combined beam propagate along *z* axis. an applied voltage bias across cell control pretilt of NLC molecules. (**c**) Original pure image. (**d**) Completely merged image.

The schematic diagram of the SR system is shown in Fig. 1(a). The 514 nm continuous laser light with polarization aligned with *x* axis is split into two beams. The signal beam is coherent light carrying information of the resolution chart (200 μm × 200 μm) with fixed intensity of 1.4 × 10^3^ W/cm^2^. The noise beam generated by passing it through a rotating diffuser (rotate speed 500 Hz) is a partially spatially incoherent light with random phase fluctuations, the correlation length of which is determined by a “lens-diffuser-lens” system. Specifically, to change the correlation length of noise we can adjust the ratio of the beam diameter focused on the diffuser to the average size of the scattering centers or speckles by translating the diffuser within the confocal telescope^14,15^. After the noise beam passes through a rotating diffuser, the degree of polarization of the noise beam will change. An analyzer is placed behind “lens-diffuser-lens” system to keep polarization align with x axis. Then two combined beams are simultaneously injected into NLC sample along *z* axis, unlike usual setups in reference^7,8^. The beam diameter of the two beams on incident plane of NLC cell is 175 μm. Finally, light exiting the NLC is imaged into a CCD camera. The NLC sample we employed is 330 μm-thick glass cell full with undoped nematic E7 (indices *n*_⊥_ = 1.53, *n*_*∥*_ = 1.77, low-frequency dielectric *ε*_⊥_ = 5.1, *ε*_*∥*_ = 19.6 and the average elastic constant *K*_*N*_ = ~10^−11^ N)^16^. The cell thickness in the z-direction is 500 μm. The PVA-coated planar interfaces anchor the NLC director with a small tilt with respect to the *z* axis, as to prevent the formation of reorientational domains and disclinations. The 1 kHz sinusoidal low-frequency bias voltage across the cell controls the pretilt angle of liquid crystal molecules in the x-z plane. An input glass interface, parallel to *x*, prevents the occurrence of undesired depolarization effects.

### Modulation instability growth rate

**Figure 2:**
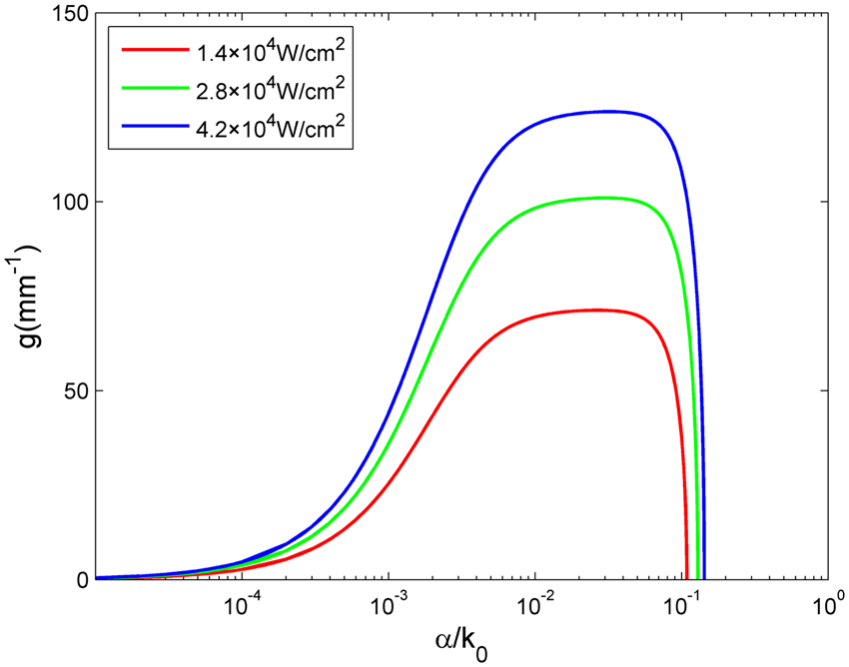
Modulation instability growth rate of perturbation mode with the pump light intensity of 1.4 × 10^4^ W/cm^2^, 2.8 × 10^4^ W/cm^2^ and 4.2 × 10^4^ W/cm^2^.

We theoretically analyze this issue mainly with modulation instability gain of perturbation modes. Figure 2 shows the modulation instability growth rate of perturbation modes with the different pump light intensity. There is a cut-off frequency that changes with the pump light intensity. And the low frequencies are barely amplified when light passes NLC. So NLC in this process can be seen as an adjustable band-pass filter with no energy loss in the ideal condition. Here, the increase of noise light treated as the pump light changes the growth rate of modulation instability in NLC. The growth rate of perturbation modes becomes large, in particular for a specific band of perturbation modes. In addition, the growth rate curve of modulation instability shifts towards higher frequencies. The optimally amplified frequency and the cut-off frequency both become higher. As a consequence, the higher frequencies are selectively amplified with the change of the modulation instability growth rate curve. Mode matching between the optimally amplified frequency of modulation instability in NLC and frequency components of the signal reaches optimum at the specific level of noise. At this moment the system is in the resonance state and the quality of signal recovery is best. Note that the matching degree of the optimally amplified frequency and main frequencies in signal determines the quality of signal recovery owing to the multi-modal nature of image signal. We note the dispersion relation about incoherent light propagation in NLC in reference^16^ is identical in form to the dispersion relation for electron plasma waves. This implies the incoherent light of background in NLC can be treated as photonic plasma^17^. We set a weak signal beam as the wave launched into photonic plasma. So by analogy, the coupling of the weak signal to the incoherent noise background can also be regarded as a typical beam-plasma interaction. According to this explanation, with the combined input light of noise and weak signal propagating in NLC, signal modes (here as perturbation modes) grow at the expense of the noise. At the specific level of noise the resonance modes of the system match signal modes, then resonance takes place, i.e. stochastic resonance. In conclusion, this is a self-filtering process of input based on modulation instability in NLC, resulting in the change of output frequency spectrum and extraction of the signal. In addition, there is no energy waste because noise energy transfers to signal rather than being filtered directly.

In the spatial domain, the weak signal seeds a potential well. Noise is trapped to the potential well, which reinforces this well. Afterwards, more noise light is concentrated into this reinforced well. The energy of noise light transfers to the signal with this lasting process, which makes the signal enhanced at the expense of noise. This explanation is similar to the gradient theory of reference^9,10^. The modulation instability gain theory and gradient theory are the frequency domain comprehension and the spatial domain comprehension of this nonlinear process, respectively. The growth of signal modes (perturbation modes) in the frequency domain corresponds to the formation of induced optical waveguide by the weak signal in the spatial domain.

### Output images and cross-correlation

To quantitatively evaluate the improvement of the similarity between input and output images, we use the cross-correlation coefficient and cross-correlation gain, which are defined as^13^

1 $${C}_{I,{I}_{0}}=\frac{\langle (I-\langle I\rangle )({I}_{0}-\langle {I}_{0}\rangle )\rangle }{{[\langle {(I-\langle I\rangle )}^{2}\rangle \langle {({I}_{0}-\langle {I}_{0}\rangle )}^{2}\rangle ]}^{1/2}}.$$

2 $${C}_{g}={C}_{{I}_{out},{I}_{0}}/{C}_{{I}_{in},{I}_{0}}.$$

In which *I*_0_, *I*_*in*_, *I*_*out*_ are the pure image, input noisy image and output image, respectively.

**Figure 3:**
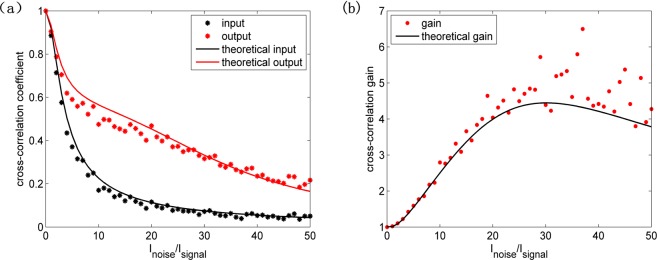
(**a**) Cross-correlation coefficient and (**b**) cross-correlation gain versus noise-to-signal intensity ratio with fixed applied voltage bias of 3.6 V.

Figure 3(a,b) depict the cross-correlation coefficient and cross-correlation gain with the noise-to-signal intensity ratio, respectively. Here, noise intensity is defined as average intensity of the speckle field on incident plane of NLC cell. Based on the theory about the modulation instability in NLC as shown in Method, the theoretical curves are obtained (NLC cell thickness 330 μm, the calculated length along z axis 500 μm, anchoring angle with respect to the *z* axis 1°, E7 property parameters: indices *n*_⊥_ = 1.53, *n*_*∥*_ = 1.77, low-frequency dielectric *ε*_⊥_ = 5.1, *ε*_*∥*_ = 19.6 and the average elastic constant *K*_*N*_ = ~10^−11^ N, applied voltage bias 3.6 V, the coherence length 170 μm). As we can see, there is a small deviation of cross-correlation coefficient compared with the theoretical curves due to perturbation of the noise beam. In particular, when noise-to-signal intensity ratio is relatively larger the intensity perturbation of the noise beam also becomes larger. By stronger coupling in NLC, this larger perturbation of the noise beam causes greater deviation, more obvious in cross-correlation gain. However, the theoretical curves basically match the results well. With the increase of noise the cross-correlation coefficient of the pure image and input noisy image decreases, compared with which the cross-correlation coefficient of the pure image and output noisy image versus noise-to-signal intensity ratio is smoother. The quality of output noisy image compared with the input noisy image is improved because a part of noise energy transfers to the signal due to SR. the positive impact of SR for image recovery enlarges with the increase of noise until the noise-to-signal intensity ratio reaches 30:1, beyond which the positive impact of SR diminishes. The peak of cross-correlation gain curve is the characteristic signature of SR.

In viewpoint of the spatial space, a potential well created by the weak signal appeals more noise and becomes stronger with the increase of noise. However, when noise is beyond the optimal value, the diffraction will be markedly enhanced, which leads to the decline of SR impact. In viewpoint of the frequency space, the increasing noise changes the growth rate of frequency. The high frequencies corresponding to the frequency components of signal are enhanced because of the selective amplification of modulation instability with the increase of noise, which promotes the impact of SR and the visibility of output image. The higher frequencies corresponding to the frequency components of noise are amplified when the noise-to-signal intensity ratio exceeds the optimal value, which weakens the impact of SR. When the noise intensity is much larger than the signal beam intensity, especially for an overwhelmed signal, the linear perturbation theory can analyze well the results.

**Figure 4:**
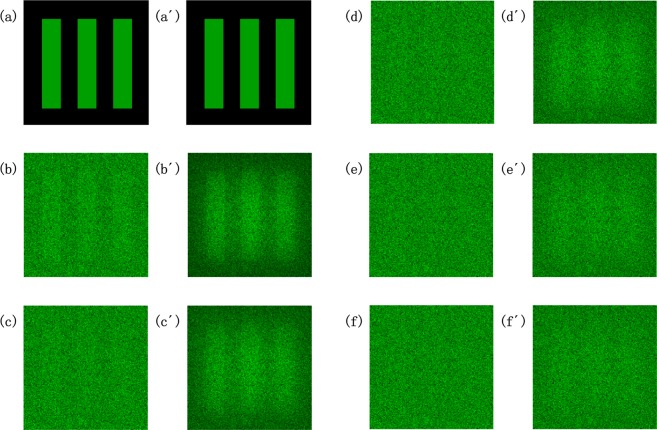
Input and output images with different noise-to-signal intensity ratio. (**a**–**f**) Input images with noise-to-signal intensity ratio of 0:1, 10:1, 20:1, 30:1, 40:1, 50:1, respectively. (**a′**–**f′**) corresponding output images to (**a**–**f**), respectively. Size of images is 175 μm × 175 μm.

Figure 4 shows images with different noise-to-signal intensity ratio. The vague input noisy image becomes explicit when intensity ratio is small as showed in Fig. 4(b,b′). The image can still be recovered even if the signal is obscured completely as showed in Fig. 4(c,c′). Capacity of noisy image reconstruction via SR is limited in the case of very high intensity ratio as showed in Fig. 4(d′,e′). It is worthy of noticing that the edges of bars are not so sharp due to smoothness of refractive index distribution in NLC.

**Figure 5:**
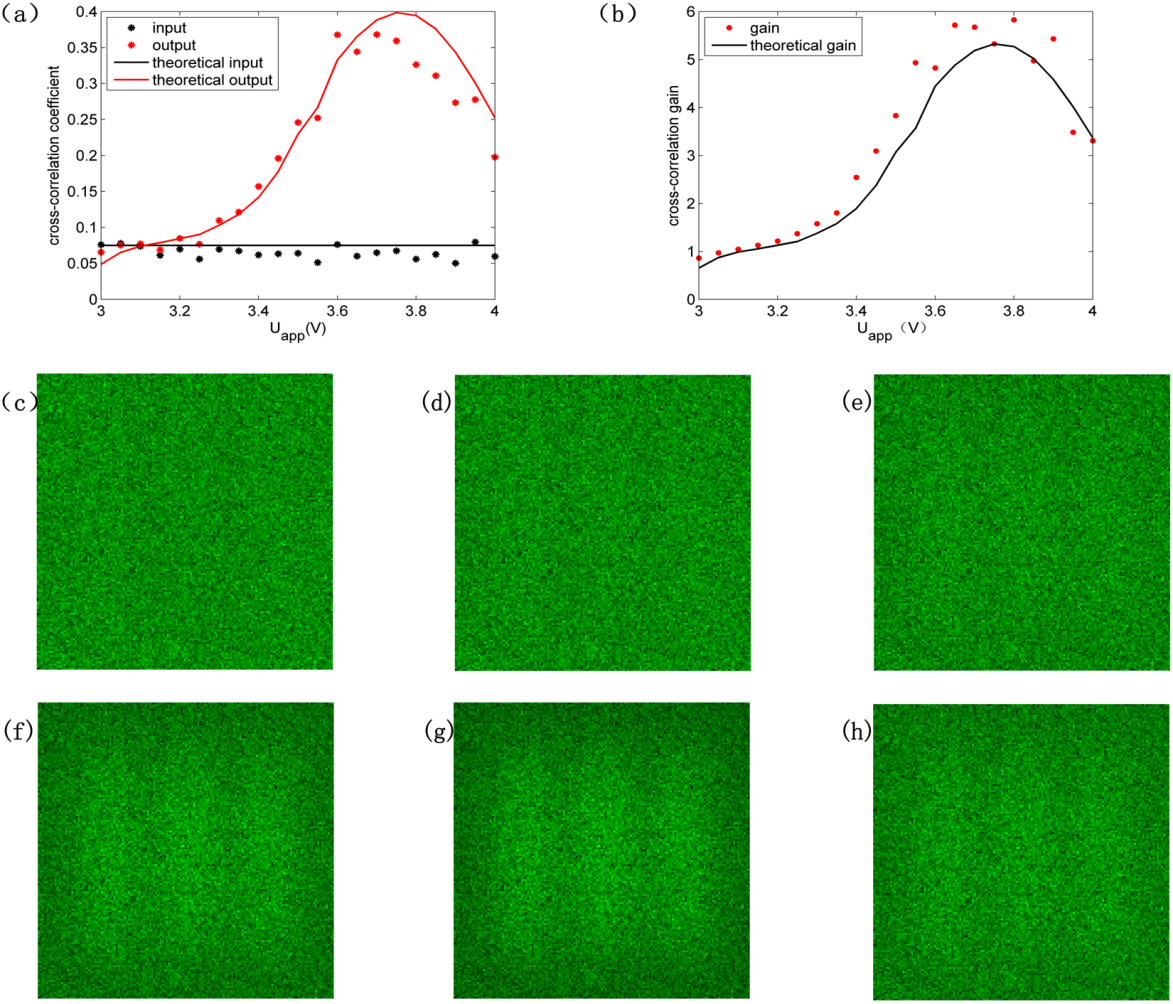
(**a**) Cross-correlation coefficient and (**b**) cross-correlation gain versus applied voltage bias with fixed noise-to-signal intensity ratio of 30:1. (**c**–**h**) are output images with applied voltage bias (V) of 3, 3.2, 3.4, 3.6, 3.8, 4. Noise-to-signal intensity ratio is 30:1. Size of images is 175 μm × 175 μm.

The cross-correlation coefficient and cross-correlation gain as the function of voltage are presented in Fig. 5(a,b), respectively. The theoretical curves are obtained with the identical parameters in Fig. 3(a,b) except that noise-to-signal intensity ratio is fixed at 30:1 and the applied voltage bias is changeable. We can see from cross-correlation coefficient that there is still the perturbation of the noise beam. When the applied voltage bias is relatively larger the coupling interaction in NLC becomes stronger, the perturbation of the noise beam causes greater deviation. When the applied voltage bias is around more suitable voltage value 3.6 V the stronger coupling makes image extraction ability better. On the whole the theoretical curves correspond with the results well. The quality of output image is promoted with the rise of voltage until voltage reaches approximate 3.8 V, beyond which the quality of output image becomes bad. The pretilt angle of liquid crystal molecules enlarges and the general characteristic length of the nonlinear nonlocality decreases with the increase of voltage bias. The growth rate of modulation instability in NLC changes and the higher frequencies will be amplified with the increase of the pretilt angle of liquid crystal molecules and decrease of the general characteristic length of the nonlinear nonlocality^18^. The quality of output image is optimum when the amplified frequencies are corresponding to the frequency components of signal. It is noise that is mainly amplified when the voltage bias exceeds the optimal value. In particular, the output image is worse than the input noisy image when voltage is below 3.1 V because very low frequencies corresponding to the frequency components of noise are enhanced. Figure 5(c–h) show the output images when NLC is applied with different voltage. The output images in Fig. 5(c–h) are almost as blurred as the input image when voltage is low. The output image becomes recognizable when voltage is approximate 3.8 V, which can be further improved by the general signal processing techniques, such as averaging and thresholding.

**Figure 6:**
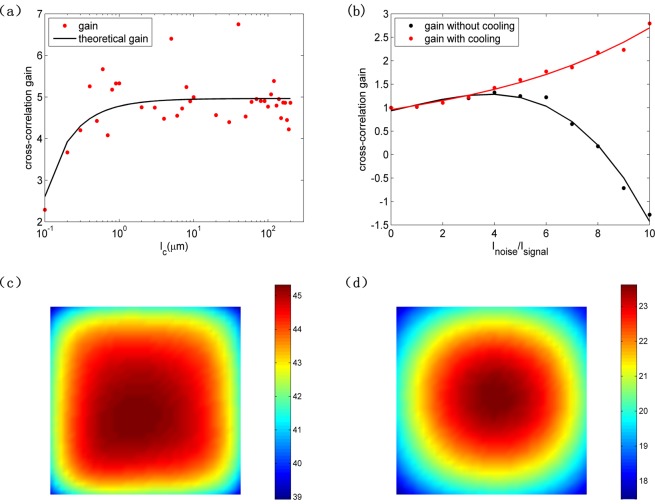
(**a**) Cross-correlation gain versus coherence length with applied voltage bias of 3.6 V. (**b**) cross-correlation gain versus noise-to-signal intensity ratio with cooling and without cooling. temperature distribution on image area of exit surface (**c**) without cooling and (**d**) with cooling. Noise-to-signal intensity ratio is 10:1.

The impact of the coherence length on cross-correlation gain is numerically calculated as shown in Fig. 6(a). The theoretical curves are obtained with the identical parameters in Fig. 3(a,b) except that noise-to-signal intensity ratio is fixed at 30:1 and the coherence length is changeable. The theoretical curve accords with the results well except some relatively large deviation dots due to the perturbation of the noise beam. The cross-correlation gain is very small when the coherence length is short. Modulation instability is a phenomenon that interaction of nonlinearity and diffraction leads to the formation of modulated pattern. For incoherence light, nonlinearity needs to balance noise statistics except diffraction. When the coherence length of noise light is so short that the noise statistics is beyond nonlinearity, the incoherence light cannot be trapped by nonlinearity and the quality of images becomes worse. Considering that the coherence length of noise light is not too long in reality, the coherence length we employed in above analysis is 170 μm.

Laser-induced thermal effect on noisy image recovery process is studied here. As we know, laser propagating in NLC will generate thermal effect and make temperature of NLC rise. As temperature increases, the extraordinary refractive index of nematic liquid crystal E7 decreases while the ordinary refractive index of E7 changes little^19^. This means that the birefringence and nonlinearity of E7 will also decrease. Molecule reorientation nonlinearity and thermal nonlinearity have the opposite effect with increase of the light intensity in E7. The thermal effect plays a negative role for noisy image recovery. So, to decrease the thermal effect we used a pure nematic liquid crystal E7, which has a weaker negligible thermal effect than the doped NLC in general. However, when laser intensity is relatively strong, laser-induced thermal effect in pure E7 cannot be also neglected. Figure 6(b) shows heat effect on cross-correlation gain. By solving Eq. (14) numerically, we obtain the curves with cooling and without cooling (thermal conductivity 0.2 W/(m∙K), absorbance 10 m^−1^, without cooling convective heat transfer coefficient 5 W/(m^2^∙K), external medium temperature 20 °C; with cooling convective heat transfer coefficient 10000 W/(m^2^∙K), external water temperature about 10 °C). As we see from Fig. 6(b), when there is no external cooling, the thermal effect is week and the extraction ability of image strengthens with increase of the noise when noise-to-signal intensity ratio is fairly small. The thermal effect exceeds molecule reorientation effect and the extraction ability of image diminishes when noise-to-signal intensity ratio is beyond 4:1. Particularly, when noise-to-signal intensity ratio is beyond 8:1 cross-correlation coefficient of the pure image and output noisy image becomes negative because the thermal effect dominates and the output image is badly destroyed. When we use an external cooling approach that NLC was cooled by a flow of water (close to 10 °C) near the interfaces of two glass plates parallel to z axis, similar to reference^20^ (not shown in Fig. 1), this approach can effectively eliminate the heat due to light absorption. Figure 6(c,d) show the temperature distribution on image area of exit surface without cooling and with cooling when noise-to-signal intensity ratio is 10:1, respectively. Without cooling temperature rises to around 40 °C, which will induce considerable thermal nonlinearity and cause negative effect on image recovery. With cooling temperature basically stays around 20 °C, which makes thermal effect negligible. In conclusion, with appropriate cooling thermal effect can be neglected and image recovery of high quality can be obtained.

## Conclusion

In this paper, we demonstrate stochastic resonance based on modulation instability in nematic liquid crystals and further develop a practical technology to reconstruct noise-hidden images. We found that the input noisy intensity, the applied voltage and the correlation length influence the quality of output image. By carefully designing these parameters hidden images information can be extracted effectively. Nematic liquid crystals provide a mature nonlinear medium for practical application of image recovery via SR. Meantime, in view of the similar nature of NLC and some other soft matters, this research inspire that this technology may be feasible in other soft matters.

## Method

The electric dipole moment parallel to the axis of rod molecule will be induced when a rod liquid crystal molecule is exposed in the optical field or electric field. The induced dipole moment tends to align with the polarization of laser light, which makes the molecule rotate. This rotary movement will be on-going until liquid crystal molecule reaches a steady state when the elastic force between molecules achieves a balance with the turning force induced by laser light. According to the birefringence model, the axis of rod molecule is the optical axis in NLC and the change of the angle between the optical axis and the polarization of laser light will affect the refractive index of extraordinary light. In turn, the change of refractive index due to reorientation of liquid crystal molecules will alter the propagation of laser light. When the applied external voltage is fairly large, the pretilt distribution of liquid crystal molecules in the light-passing area is fairly flat. The director distribution of molecules can be expressed as^21^

3 $${\nabla }^{2}{\rm{\Delta }}\theta -\frac{1}{{\omega }_{m}^{2}}{\rm{\Delta }}\theta +{\varepsilon }_{0}{\varepsilon }^{op}\langle {{\rm{\Psi }}}^{\ast }{\rm{\Psi }}\rangle \sin \,2{\theta }_{0}/4{K}_{N}=0.$$

where *∆θ* is the change of director due to laser light, *ε*^*op*^ (*ε*^*op*^ = *n*_*∥*_^2^ − *n*_⊥_^2^) is the birefringence, *ε*_0_ is the permittivity of vacuum, Ψ is the amplitude of laser light, *θ*_0_ is the pretilt controlled by the electric field. When the rotation rate of diffuser is sufficiently high, the response time of NLC is much longer than the characteristic time of intensity fluctuations. Due to the noninstantaneous nature of NLC response, we just consider the time-averaged intensity distribution in the spatial space. The bracket denotes a time average for intensity term^22^. *ω*_*m*_ is the general characteristic length of the nonlinear nonlocality, described as^23^

4 $${\omega }_{m}=\frac{1}{E}{[\frac{2{\theta }_{0}{K}_{N}}{{\varepsilon }_{0}{\varepsilon }^{rf}\sin 2{\theta }_{0}-2{\theta }_{0}{\varepsilon }_{0}{\varepsilon }^{rf}\cos 2{\theta }_{0}}]}^{\frac{1}{2}}.$$

where *E* is the average electric field, *ε*^*rf*^ (*ε*^*rf*^ = *ε*_*∥*_ − *ε*_⊥_) is the low-frequency anisotropy. Here, pretilt *θ*_0_ and *E* depend on the voltage bias^24^

5 $$V({\theta }_{0})=2\sqrt{\frac{{K}_{N}(1+{\sin }^{2}{\theta }_{0}{\varepsilon }^{rf}/{\varepsilon }_{\perp })}{{\varepsilon }_{0}{\varepsilon }^{rf}}}{\int }_{0}^{{\theta }_{0}}\frac{d\theta }{\sqrt{({\sin }^{2}{\theta }_{0}-{\sin }^{2}\theta )(1+{\sin }^{2}\theta {\varepsilon }^{rf}/{\varepsilon }_{\perp })}}.$$

6 $$E=\frac{V{\int }_{0}^{{\theta }_{0}}\sqrt{\frac{1+{\sin }^{2}\theta {\varepsilon }^{rf}/{\varepsilon }_{\perp }}{{\sin }^{2}{\theta }_{0}-{\sin }^{2}\theta }}d\theta }{H(1+{\sin }^{2}{\theta }_{0}{\varepsilon }^{rf}/{\varepsilon }_{\perp }){\int }_{0}^{{\theta }_{0}}\frac{d\theta }{\sqrt{({\sin }^{2}{\theta }_{0}-{\sin }^{2}\theta )((1+{\sin }^{2}\theta {\varepsilon }^{rf}/{\varepsilon }_{\perp })}}}.$$

where *H* is the thickness of liquid crystal sample.

Ignoring the impact of liquid crystal boundary, Eq. (1) has the solution in the form^23^:

7 $${\rm{\Delta }}\theta =\frac{{\varepsilon }_{0}{\varepsilon }^{op}\,\sin \,2{\theta }_{0}{\omega }_{m}^{2}}{4{K}_{N}}{\int }_{-\infty }^{\infty }R({\boldsymbol{r}}-{\boldsymbol{r}}{\boldsymbol{^{\prime} }})\langle {{\rm{\Psi }}}^{\ast }{\rm{\Psi }}\rangle d{\boldsymbol{r}}{\boldsymbol{^{\prime} }}.$$

and

8 $$R({\boldsymbol{r}})=\frac{1}{2\pi {\omega }_{m}^{2}}{K}_{0}(\frac{\sqrt{{x}^{2}+{y}^{2}}}{{\omega }_{m}}).$$

where *K*_0_ is the zeroth order modified Bessel function.

The nonlinear propagation of light in NLC is described by Fock-Leontovich equation^21^

9 $$2ik\frac{\partial {\rm{\Psi }}}{\partial z}+{\nabla }^{2}{\rm{\Psi }}+{k}_{0}^{2}{\varepsilon }^{op}{\rm{\Delta }}\theta \,\sin \,2{\theta }_{0}{\rm{\Psi }}=0.$$

with *k*^2^ = *k*_0_^2^[*n*_⊥_^2^ + *ε*^*op*^ sin^2^*θ*_0_], *k*_0_ = *ω*/c.

In NLC, there is a nonlinear coupling based on modulation instability, which is related to diffraction and nonlinearity^25^. We adopt the Wigner-Moyal transform method. When the noise intensity is much larger than the signal beam intensity, the linear perturbation theory can be used. the Wigner distribution of the light can be described:

10 $$f({\boldsymbol{r}},{\boldsymbol{k}},z)={f}_{0}({\boldsymbol{k}})+{f}_{1}({\boldsymbol{k}})\cdot \exp (gz)\cdot \exp (i{\boldsymbol{\alpha }}\cdot {\boldsymbol{r}}).$$

and

11 $$\langle {{\rm{\Psi }}}^{\ast }{\rm{\Psi }}\rangle ={\int }_{-\infty }^{\infty }{d}^{3}{\boldsymbol{k}}f({\boldsymbol{k}}).$$

For simplicity, we reduce the problem to one dimension. The generalized dispersion relation can be written as^16^

12 $$0=1+\frac{{C}_{1}{C}_{3}}{{K}_{N}\cdot {\alpha }^{2}+{C}_{2}}{\int }_{-\infty }^{\infty }dk\frac{{f}_{0}(k+\alpha /2)-{f}_{0}(k-\alpha /2)}{-ig+\alpha \beta k}.$$

In the case of a Lorentzian distribution of incoherent noise spatially uniform distribution ƒ_0_(*k*), the growth rate of modulation instability can be obtained in closed form^16^:

13 $$g=\alpha \beta \cdot \sqrt{\frac{{C}_{1}{C}_{3}{A}_{0}^{2}}{\beta ({K}_{N}\cdot {\alpha }^{2}+{C}_{2})}-\frac{{\alpha }^{2}}{4}}-{\rm{\Delta }}k\alpha \beta .$$

for a perturbation mode with wavenumber *α*, where *β* = *λ*/2π*n*_0_ is the diffraction coefficient of wavelength *λ*, *n*_0_ is the base index of refraction, *C*_*1*_ = *ε*_0_*ε*^*op*^/4sin(2*θ*_0_), *C*_*2*_ = *ε*^*rf*^*E*^2^[1 − 2*θ*_0_cot(2*θ*_0_)]sinc(2*θ*_0_), *C*_*3*_ = *k*_0_*ε*^*op*^sin(2*θ*_0_)/*n*_0_, *A*_0_ is the amplitude of laser light, *Δk*~1/*l*_*c*_, *l*_*c*_ is the coherence length of incoherent noise. We note that the Gaussian distribution is truer for the spatially incoherent beam we used. However, when we take the angular power spectrum of the incoherent beam as a Lorentzian distribution, the evolution of incoherent modulation instability is similar to the Gaussian case in the initial stage. Also, assuming that the noise has a Lorentzian distribution, we can get a closed-form solution of the growth rate of modulation instability^16,26^. So we adopt this simple model and the model can basically explain the real case.

When laser intensity is relatively strong, laser-induced thermal effect cannot be neglected in general. The thermal response of nematic liquid crystal E7 is very fast compared with the molecule reorientation nonlinearity response, so we just consider the steady-state case of heat transfer in E7. The steady-state heat transfer equation on light-passing surfaces can be written as

14 $${\rm{\Delta }}T(x,y)=-\frac{\alpha }{\kappa }I(x,y)$$

where ∆ is Laplace operator, *κ* is the thermal conductivity, *α* is the absorbance.

The boundary condition in our setting can be described:

15 $${[T(x,y)+\frac{\kappa }{h}\cdot gradT(x,y)]}_{x={x}_{0},y={y}_{0}}={T}_{0}$$

where *h* is the convective heat transfer coefficient, x_0_ and y_0_ are boundary coordinates, *T*_0_ is the external medium temperature.
